# Reproductive health in the recent disasters of Iran: a management perspective

**DOI:** 10.1186/s12889-018-5311-2

**Published:** 2018-03-21

**Authors:** Sanaz Sohrabizadeh, Katayoun Jahangiri, Reza Khani Jazani

**Affiliations:** Department of Health in Disasters and Emergencies, School of Health, Safety and Environment, Shahid Beheshti University of Medical Sciences, Tehran, Iran

**Keywords:** Reproductive health, Disaster, Management, Iran

## Abstract

**Background:**

Natural disasters represent critical threats to the health, safety, and well-being of a community—including reproductive health, which has been neglected in disaster-stricken regions. The current study was undertaken to explore administrative issues related to post-disaster reproductive health systems in Iran.

**Methods:**

A qualitative approach using in-depth unstructured interviews was applied to explore the administrative issues of reproductive health systems in the affected regions. A total of 22 participants were interviewed using the purposeful sampling method. Data were collected in three provinces: East Azerbaijan, Bushehr, and Mazandaran. Transcribed interviews were analyzed using the conventional content analysis.

**Results:**

Six categories of management issues of reproductive health in disasters were extracted from data. These categories were: ignoring cultural factors, lack of planning, lack of training, insufficient data collection, ignoring male reproductive health, and lack of monitoring systems.

**Conclusion:**

Different challenges to reproductive health management that emerged from the research should be considered and included in reproductive health plans and policies in disaster-affected regions in Iran. Involving community in all processes of providing reproductive health services, from planning to monitoring, is highly suggested.

## Background

Natural disasters represent critical threats to the health, safety, security, or well-being of a community. On the other hands, management issues can exacerbate the health consequences of natural disasters. Coordination among disaster management partners, developing information management system, community-based interventions, close collaboration of relevant organizations, building the culture of disaster prevention, logistic and supply management systems, national disaster management plan as well as monitoring and assessment of disaster management interventions include neglected disaster management issues of health systems in Iran [1].

Humanitarian emergencies resulting from natural disasters have important health complications, including reproductive health issues [2]. Reproductive health has historically been neglected in disaster-affected settings [3]. Reproductive Health (RH) is defined as a state of complete physical, mental, and social well-being (not merely the absence of disease and infirmity) in all matters relating to the reproductive system and its functions and processes [4]. RH implies that women and men are capable of having a satisfying and safe sex life as well as having the right to be informed and to have access to safe, effective, and affordable family planning methods based on their choices [5].

Disasters can increase vulnerability to poor reproductive health outcomes among affected populations due to reduced access to RH services and supplies, destroyed health facilities, insufficient human resources, exposure to sexual violence, and impoverishment [6]. Accordingly, there is evidence of high rates of unmet needs and lack of investment in RH services after disasters [7, 8]. For example, the rates for pelvic inflammatory disease, lower genital tract infections, and menstruation disorders increased after the massive Wenchuan earthquake [9]. Furthermore, the study of risk factors for depression and anxiety in women with an earthquake experience reported that a significant proportion of differences in the experience of depression resulted from post-earthquake reproductive health issues with poverty, insufficient family support, and lack of access to health care facilities [10].

The responsibility of health authorities to respond to the RH needs of disaster-affected people has been increasingly recognized over the past two decades [11]. For example, the Inter-Agency Field Manual on Reproductive Health in Humanitarian Settings was developed in 1999 by the Inter-Agency Working Group for Reproductive Health in Refugee Settings (IAWG) and World Health Organization (WHO) to acknowledge the reproductive health needs of disaster-affected people [12]. The manual addresses Minimum Initial Service Package (MISP), family planning, HIV/AIDS, and sexually transmitted diseases (STDs) among disaster-affected populations [5].

Regarding the performance of global guidance including Inter-Agency Field Manual on Reproductive Health in Humanitarian Settings, global evaluations showed both progress and gaps in providing reproductive health services in crises. For instance, awareness and funding for reproductive health have increased significantly, and provision of service has expanded. On the other hand, lack of financial support and systematic implementation as well as variable quality, program inefficacy are the examples of gaps in reproductive health services in different contexts. Furthermore, poor commodity management and security, limited availability of abortion care, and lack of community mobilization to increase reproductive health service reported as critical gaps [12, 13].

On the other hand, addressing the reproductive health needs and rights of affected women, men, and adolescents is crucial to ensuring their well-being in highly disaster-prone countries [14], and to achieving the Sustainable Development Goals (SDGs) until 2030 [15]. Disasters are frequently characterized by the collapse of healthcare infrastructure [2]. Changes in post-disaster reproductive healthcare can increase unwanted pregnancy, STDs, pregnancy complications, and maternal mortality [16]. Provided health services varies depending on location, culture, existing healthcare infrastructure, health of population, type of disasters and multiple humanitarian organizations [2–17].

In the Iranian context, despite the negative effects of previous disasters on reproductive health systems, little research has been conducted on the administrative challenges of providing reproductive health services after disasters. Instead, considerable numbers of research papers have focused on negative effects of disasters on reproductive health of affected people. For example, the reproductive health indicators of the women affected by the twin quakes of East Azerbaijan (2012) indicated a decrease of live birth rate, general marriage fertility rate, contraceptive methods coverage and increase of stillbirth rate [18]. In addition, a qualitative study of the consequences of earthquakes on women’s reproductive health reported four main consequences: psychological complications, reproductive system physical damages, sexual health damages, and fertility regulation [19].

While the evidence on reproductive health in stable settings is extensive, the literature regarding reproductive health management issues after disasters is less well explored and documented. To fill this gap, the current study was undertaken to explore administrative issues related to post-disaster reproductive health functions in Iran. Identifying the reproductive health management challenges in disasters can lead to improving the current reproductive health systems, which will in turn lead to being effectively prepared for future destructive disasters.

## Methods

### Study design

A qualitative approach using conventional content analysis was used to obtain insight into the experiences and views of participants as well as identifying additional relevant variables and new research questions [20]. With this approach, data is gathered directly without any previous hypothesis; then, codes, sub-categories, and categories are extracted by the inductive process [21].

### Setting

Iran is a highly hazard-prone country frequently affected by natural disasters such as earthquakes, flood, and drought [22]. This country has a population of over 70 million with almost equal gender distribution (49.6% women). Among literate women (more than 80%), about 18.4% have had a formal academic education, and 12.1% of households are headed by women [23].

Health services are provided through a nationwide network which consists of a referral system starting at primary care centers in rural areas and moving through secondary and tertiary hospitals in the provincial capitals. The public sector provides primary, secondary, and tertiary health services, and is the main provider of primary health care services, including reproductive health of women. Consequently, the coverage of reproductive health services in rural areas and small cities is 100%. Behvarz are community health workers (rural women and men) who provide primary health care services at rural levels of Iran’s health network [24].

This study was conducted in three provinces of East Azerbaijan, Bushehr, and Mazandaran, which experienced destruction by earthquakes and floods between 2012 and 2013. The earthquakes and floods killed more than 350 citizens and injured more than 3000 people living in the affected regions [10]. In addition, more than 70% of all victims were women and children [25].

### Participants

Key informants and health workers were approached for interviews. Health workers provided reproductive health services in the affected fields of East Azerbaijan, Bushehr and Mazandaran. The key informants included professors and scholars of reproductive health who had experiences in some disaster-affected regions as health care providers. That is, they considered as key informants based on their knowledge as well as experiences on the subjects of reproductive health in disasters. The criteria for inclusion were having experience of at least one natural disaster and a willingness to participate in the study.

A purposeful sampling method was used to select participants for interviews. Health workers who represented the experiences of providing reproductive health services in the affected regions and key informants who had both knowledge and experiences on reproductive health in disasters were approached for interviews. The number of participants was determined based on saturation principles: that is, until the quality and amount of information were sufficient and no new concepts emerged from data. Data saturation was reached after 21 interviews; however, one additional interview was conducted to make certain that new concepts were not developed. A total of 22 participants, including 12 health workers as well as 10 key informants, were interviewed.

### Data collections

A purposive sampling method was used to collect data. Data were gathered through face-to-face and unstructured, in-depth interviews which were performed in the affected regions (East Azerbaijan, Bushehr, and Mazandaran) and at key informants’ offices. Each interview lasted between 50 and 90 min. The researcher prompted each participant: “Explain how was your experience as a reproductive health provider after the quakes or floods.” Probing was done to motivate the respondents to describe their experiences and perceptions. All interviews were performed, audio recorded, and transcribed verbatim in Farsi and confirmed by the participants. Each interview was analyzed immediately and the retrieved information became a guide for further data collection.

### Data analyses

A qualitative content analysis using the Graneheim approach was applied for data analysis [21]. Accordingly, several steps were conducted to analyze the data.

First, the interviews were read several times to get a sense of the whole. Second, meaning units were identified and highlighted. Third, the meaning units were condensed into shorter units. Fourth, the condensed meaningful units were abstracted and labeled with a code. At this stage, the quality control of coding was done by peer checking. Finally, the various codes were compared according to differences and similarities and sorted into six categories. The process was recursive, with the author moving back and forth between the stages of concept development.

### Trustworthiness

The strategies of credibility, dependency, confirmability, and transferability were used for achieving data trustworthiness. Triangulation strategies, such as research field triangulations and including both earthquakes and floods as well as member and peer checking, were applied for achieving credibility and dependency criteria. Regarding member checking, the researcher asked the participants about possible misunderstandings during interviews. In peer checking, the data analysis process was monitored independently by one researcher who was not involved in the research process. The resulted codes were compared with the codes which had been extracted by the researchers to elicit the final concepts. Confirmability and transferability of data were provided by a detailed description of the process of the study.

## Results

The coverage rate of the post-disaster reproductive health services in the affected regions of east Azerbaijan were available in the Ministry of Health (Table 1). These data can support the necessity of performing a qualitative research in order to retrieving the main factors which negatively influence reproductive health management in the destroyed regions.

**Table 1:** Coverage rate of reproductive health services in the affected regions of east Azerbaijan (August and September 2012)

Reproductive health services	^a^Coverage (%)	
	Pre-earthquake	Post-earthquake
Pregnant mothers	100	55.7
High risk mothers	100	69.7
Women in reproductive age	100	18.9
Care for under 1 year children	100	30.5
Care for 1-5 year children	100	29.9

Source: Population Health Office, Ministry of Health, Iran, 2017

^a^Coverage of reproductive health services in rural areas and small cities is 100% in the reason of Primary Health Care networks in Iran. This network have brought the primary health care to all remote villages and cities pre-disasters

The participants were between 25 to 55 years old, mostly between 45 and 55 years (45%) (Table 2). Of these, 64% were female (10 health personnel and 4 key informants), and the remaining 36% were male (5 health workers and 3 key informants). The educational level ranged from Behvarzi diploma (27%) to academic education (73%).

**Table 2:** Demographic information of the participants

Variable		n	n%
Gender			
	Female	14	64
	Male	8	36
Age (year)			
	25-35	5	23
	35-45	7	32
	45-55	10	45
Education level			
	Diploma (Behvarzi)	6	27
	Academic education	16	73

The demographic information of the participants has been illustrated based on gender, age, and education

The categories of ignoring cultural factors, lack of training, lack of planning, insufficient data collection, ignoring male reproductive health and lack of monitoring system were extracted as the administrative issues of meeting reproductive health needs in disasters.

### Ignoring cultural factors

Women were more willing to share their reproductive health problems with female health-care providers such as midwives and public health experts. However, providing health care services by some male community health workers (Behvarz), kept women from talking about some reproductive health issues such as menstruation disorders, pregnancy, contraceptive methods and sexually transmitted diseases. A health worker said that:

*“Some health workers are men and it should not be like this! Because women are not convenient to talk to them about their reproductive health problems. Actually, men have not welcomed for providing reproductive health services for affected women.”* (P3).

On the other hand, men were preferred to provide health services in the disaster-affected regions; however, they focused on the environmental health measures as well as preventing communicable diseases in the studied fields. A midwife said that:

“*Some affected women who had reproductive health needs just waited for a female health worker for meeting their needs or solving their reproductive health problems*” (P19).

### Lack of training

Women did not receive enough education regarding their reproductive health challenges, especially sexual violence and sexually transmitted diseases. While post-disaster conditions put the reproductive health of adult and young women at greater risk, they were not trained well enough to consider their reproductive health matters. For example, urinary and genital tract infections have increased among disaster-affected women. A midwife who provided reproductive health for affected women stated that:

*“After the quakes, the urinary and genital infections have increased among the affected women and girls in these regions. They were not trained to take such infections seriously…*” (P6).

In addition, local health care providers were not trained to cope with the cases of sexual violence in the affected regions. Thus, both local health care providers and disaster-affected people required special training on the reproductive health issues in the post-disaster phase. For instance, a health worker said that:

*“We have received no education on how to respond to sexual violence cases so far… we do not know how this case should be dealt with…”* (P10).

### Lack of planning

There were no pre-disaster planning for providing post-disaster reproductive health services at rural and urban regions. Health care providers were not assigned for doing predefined tasks in the affected fields. For example, some midwives were responsible for giving medicine to people while the reproductive health unit required their professional services post-disaster. A midwife said that:

*“We are supposed to provide reproductive health services! Reproductive health needs of affected people should be met by midwifery profession*!” (P16).

In addition, health centers faced a lack of equipment, materials, and human resources because there was no post-disaster planning for identifying the priorities and then allocating limited resources to meet the highly prioritized needs. Lack of coordination between local, regional, and national health systems which was reported by health workers can be considered as the result of no reproductive health planning in disasters. A public health expert stated that:

*“Planning for post-disaster reproductive health services? No, there have not been such plans. We have provided services in destroyed regions by ourselves…”* (P2).

### Insufficient data collection

The numbers of pregnant women were the main data that were collected after disasters. No data were gathered based on different stages of men and women’s lives. For example, there were no data related to the number of pubescent boys and girls, breastfeeding mothers, youths, adults, children, and the elderly. Collecting reproductive health data based on the different age and gender groups of people could assist decision makers with providing appropriate and needed services after disasters. A reproductive health scholar said that:

*“Making appropriate decision needs sex-disaggregated data collection. However, such data gathering has been neglected so far!”* (P11).

Data related to the reproductive health of affected women were written in notebooks prepared by different health personnel in the destroyed fields. A community health worker stated that:

*“We have no access to pre-disaster data on reproductive health and just designed notebooks for documenting some reproductive health services we have provided in the fields…”* (P15).

### Ignoring male reproductive health

Although the male population had reproductive health needs, all services were defined for women. Men were totally overlooked in reproductive health discussions and the focus was just on women with special needs. On the other hand, men had no intention of talking about any reproductive health subject and avoided such discussions. A reproductive health expert stated that:

*“Male reproductive health issues have been usually ignored pre- and post-disasters. Men’s health issues need to be addressed after disasters.”* (P1).

In addition, important subjects such as sexually transmitted diseases and effective contraception were discussed with married women in the absence of their husbands. For example, an increase of unwanted pregnancy is a reproductive health challenge after disasters and it can be prevented by partners’ decisions. Neglecting men’s well-being by health authorities as well as inappropriate social, mental, and economic situations imposed by disasters endangered men’s lives. A health worker who provided reproductive health services in the field asked:

*“Reproductive health for men!? Reproductive health is usually provided for women. Is this for men too?”* (P18).

### Lack of monitoring system

Monitoring functions were limited to pregnancy care for women who lived in the destroyed regions. No monitoring system was defined and organized to follow the reproductive health needs and challenges of the affected people. Such monitoring system can monitor reproductive health interventions in disaster-affected regions as well. Monitoring the reproductive health status of the affected women and men was more important because of the negative effects of disasters on the mental and physical health of the population. Thus, developing a system for regular monitoring of reproductive health status of the people could prevent related infectious diseases and death after disasters. A community health worker said that:

*“We have monitored pregnant women after the quakes. Women with serious problems come here for receiving health care but we won’t follow them anymore…”* (P 20).

Some women, especially in female-headed households, did not leave their settlements to take care of their children and thus, they did not receive healthcare and their reproductive health needs were ignored in the absence of a system for monitoring their health status. A reproductive health expert stated that:

*“Health system should monitor the reproductive health status of the affected people actively. Because of post-disaster issues, affected people do not pay attention to their health problems.”* (P7).

## Discussion

The present research was one of the first field studies focusing on reproductive health management in the affected regions of Iran. The findings indicated that the subjects of ignoring cultural factors, lack of training, lack of planning, insufficient data collection, ignoring male reproductive health, and lack of monitoring system influenced reproductive health management in the destroyed regions.

The results revealed that some cultural factors impacted receiving reproductive health services by women and girls. For example, women did not talk about their sexually transmitted diseases, pregnancy, and menstruation disorders to male health workers. This cultural issue rooted in the religious belief of disaster-affected people who followed Islam as their religion [26]. For example, Islamic rules demand that women avoid close interaction with men and insist on providing separate spheres for men and women [23]. On the other hand, regarding the male-dominant structure of disaster management system in Iran, men’s skills and capabilities were focused upon and men were preferred for handling different affairs of the disaster-affected communities including health issues [27]. Thus, Islamic beliefs as well as male-dominant post-disaster environment might limit the demands of women for reproductive health services in the affected regions. Regarding this finding, United Nations Population Fund (UNFPA) proposed the “culture lens” programming tool that helps policy makers analyze and understand the cultural values and structures in the management process [28]. Similarly, cultural lens can be applied in reproductive health management of disaster-affected regions.

According to the findings, women and girls were not trained for being sensitive to their reproductive health needs. On the other hand, health workers were not trained and prepared for dealing with sexual violence or infectious cases as well. A number of studies have highlighted the positive effects of education on reproductive health management in humanitarian emergencies [7, 29]. Disasters may provide the opportunity of training and educating people about the reproductive health issues that they had not known about or experienced before disasters [11]. For example, training programs on preventing reproductive health disorders such as unwanted pregnancy, sexually transmitted infections, sexual violence, and family planning after disasters can be more accepted by affected people rather than pre-disaster phases. In addition, it is important to plan training programs for all affected men and women in different age groups. Health care providers needed more education on how to be prepared to cope with sexual violence cases in the recovery phase because sexual violence may increase after disasters, and no one can support and assist victims but health workers [25].

The findings revealed that there was no planning for providing reproductive health services in the affected regions. Assigning health personnel tasks, as well as providing access to necessary facilities and equipment, required a well-defined plan. Several studies reported the importance of pre-disaster planning to provide effective reproductive health services in the affected regions [30–32]. It seems that the increase of genital and urinary infection cases in the affected regions was due to the lack of a pre-disaster plan to meeting the reproductive health needs of the people.

Based on the findings, data were not collected based on the characteristics of the affected population. For example, there were no age or sex-disaggregated databases to provide information on pubescent boys and girls, pregnant and breastfeeding mothers, and the elderly. Post-disaster sex-disaggregated data and gender analysis information could be used as the key inputs for appropriate reproductive health management [33]. A number of studies reported that lack of reproductive health databases, especially that sex-disaggregated data, had adversely impacted providing reproductive health services in the disaster-affected regions [34, 35]. For instance, there are no database or surveillance systems to report gender-based violence in Iran [25]. Thus, it may not be possible to make a suitable decision on reducing sexual violence during pre- and post-disaster phases. It seems that an electronic open access database needs to be developed for saving daily reproductive health data before and after disasters. This database can provide information for reproductive health managers and decision-makers in every time and place, and help high coverage of reproductive health services in the disaster-affected regions.

The findings indicated that reproductive health services were provided for only women in the affected regions. Although men played important roles in family planning, contraception methods, STDs, and sexual violence, their reproductive health needs were not considered by health care providers. That is, reproductive health services have been organized based on women’s needs. Several studies highlighted the key roles of men in achieving the reproductive health goals around the world [36, 37]. On the other hand, in addition to the reproductive health challenges, men faced serious mental and physical health problems due to the adverse effects of disasters [38]. A number of case studies discussed the post-disaster health problems of men in the affected regions [39, 40]. However, the post-disaster reproductive health challenges of men have not been documented well. Men may make decisions for reproductive health management at local and national level, but they deny their own reproductive health.

Based on the findings, people with reproductive health needs in the affected regions were not identified and monitored. For instance, reproductive health services were not provided for some women who could not leave their children or the elderly alone in the settlements. A number of studies reported that reproductive health services were not available and accessible for the affected people, including the groups with less mobility, such as widows, the elderly, and disabled people [9, 11, 41, 42]. Developing an active monitoring system to follow the reproductive health status of all affected people in destroyed regions may increase the effectiveness and coverage of reproductive health services and improve people’s well-being after disasters. Furthermore, all reproductive health interventions should be monitored by such monitoring systems.

Transportation difficulties in the affected areas were one limitation during data gathering. The experiences of health personnel with reproductive health services in the affected regions may not be representative of reproductive health management issues in all disaster-stricken regions. Thus, the results may not be generalizable and comparable to studies which have used assessment tools rather than unstructured interviews. In addition, lack of documentation of reproductive health data after disasters was another limitation of the study.

## Conclusion

The findings suggest that the reproductive health of the affected people can be improved through considering cultural factors, planning, training, data collection, a monitoring system, and attention to the reproductive health of men. In line with the international strategies and policies of reproductive health [5, 28, 43], the health systems of Iran needs national and local plans to meet the reproductive health needs of people at the time of disasters and emergencies. In addition, access to essential sexual and reproductive health care information and provision of services without discrimination should be included in the plans. Considering cultural and religious factors can be the key element for effective reproductive health management in the context of Iran with various ethnic groups.

Disasters can be considered an opportunity for improving the reproductive health management in the disaster-stricken fields. Involving community in all processes of providing reproductive health services, from planning to monitoring, is highly suggested. Furthermore, using electronic databases and monitoring systems which are comprehensive and available during pre- and post-disaster phases can be necessary for managing reproductive health facilities. The findings also suggest that men’s reproductive health should be mainstreamed in the plans and training programs of health systems before and after disasters.

## Abbreviations

IAWG: Agency working group for reproductive health in refugee settings
MISP: Minimum initial service package
RH: Reproductive health
SDGs: Sustainable development goals
STDs: Sexually transmitted diseases
UNFPA: United Nations population fund
WHO: World Health Organization
